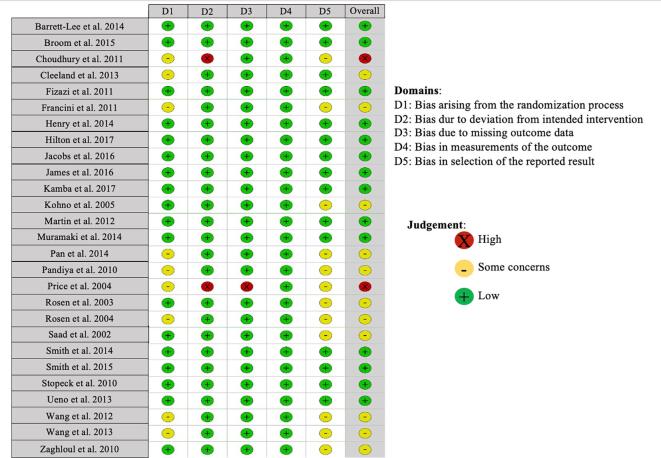# Management of bone metastasis with zoledronic acid: A systematic review and Bayesian network meta-analysis

**DOI:** 10.1016/j.jbo.2023.100470

**Published:** 2023-02-09

**Authors:** Justin-Pierre Lorange, Jose Ramirez Garcia Luna, Frédéric Grou-Boileau, Derek Rosenzweig, Michael H. Weber, Elie Akoury

**Affiliations:** a Faculty of Medicine, McGill University, Montreal, Quebec, Canada; b Department of Surgery, Division of Orthopaedics, McGill University and The Research Institute of the McGill University Health Centre, Injury Repair Recovery Program, Montreal, Quebec, Canada

**Keywords:** Zoledronic acid, Zoledronate, Bone metastasis, Clinical outcomes, Network *meta*-analysis, Adjuvant therapy, And neoadjuvant therapy

## Abstract

**Background:**

While considered the mainstay of treatment for specific bone metastases, ZA is used predominantly to treat osteolytic lesions. The purpose of this network *meta*-analysis is to compare ZA to other treatment options in its ability to improve specific clinical outcomes in patients with bone metastases secondary to any primary tumor.

**Methods:**

PubMed, Embase and Web of Science were systematically searched from inception to May 5th, 2022. Keywords used were solid tumor, lung neoplasm, kidney neoplasm, breast neoplasm, prostate neoplasm, ZA and bone metastasis. Every randomized controlled trial and non-randomized quasi-experimental study of systemic ZA administration for patients with bone metastases and any comparator were included. A Bayesian network *meta*-analysis was done on the primary outcomes including number of SREs, time to developing a first on-study SRE, overall survival, and disease progression-free survival. Secondary outcome was pain at 3, 6 and 12 months after treatment.

**Results:**

Our search yielded 3861 titles with 27 meeting inclusion criteria. For the number of SRE, ZA in combination with chemotherapy or hormone therapy was statistically superior to placebo (OR 0.079; 95 % CrI: 0.022–0.27). For the time to the first on study SRE, the relative effectiveness of ZA 4 mg was statistically superior to placebo (HR 0.58; 95 % CrI:0.48–0.77). At 3 and 6 months, ZA 4 mg was significantly superior to placebo for reducing pain with a SMD of −0.85 (95 % CrI:-1.6, −0.0025) and −2.6 (95 % CrI:-4.7, −0.52) respectively.

**Conclusions:**

This systematic review shows the benefits of ZA in decreasing the incidence of SREs, increasing the time to the first on-study SRE, and reducing the pain level at 3 and 6 months.

## Highlights

• The outcomes of zoledronic acid were assessed through a Bayesian *meta*-analysis.
• Zoledronic acid reduces the incidence of skeletal-related events and pain.
• No statistically significant treatment was found for overall survival.

## 1 Introduction

Over the past decades, oncology patients have seen their prognoses improve with new revolutionary therapies [1], [2]. Nevertheless, metastases are responsible for 90 % of cancer-related deaths [3], [4]. Along with liver and lung, bone is the most common site of metastases for many primary solid tumors such as prostate, lung, breast and kidney [5], [6], [7].

Bone metastases markedly disrupt normal bone remodelling [8]. This disturbance often results in skeletal pain, hypercalcemia and skeletal-related events (SREs) [9], [10]. SREs are a group of symptoms which comprise pathologic fractures, spinal cord compression, or events requiring radiation therapy or surgery to a bone. SREs are known for reducing physical capacity, prolonging hospital stays and shortening survival [11], [12]. Approved therapeutic agents for skeletal metastases include monoclonal antibodies such as denosumab, hormone therapy, radiotherapy, radioligand therapy, chemotherapy, immunotherapy and bisphosphonates (BPs) [13], [14].

Bisphosphonates are potent inhibitors of osteoclast-mediated bone resorption that are commonly prescribed for the treatment of osteoporosis, hypercalcemia of malignancy and bone metastases [15]. Zoledronic acid (ZA) is an intravenous, nitrogen-containing, third-generation bisphosphonate which was found to treat osteolytic lesions in vivo[13] in addition to its anti-tumor properties in vitro [16], [17], [18], [19], [20]. Moreover, ZA has been proven to be involved in inducing tumor cell apoptosis[17], modulating the immune system[20], inhibiting tumor invasion[18], decreasing tumor proliferation[19] and reducing tumor angiogenesis [16].

Therefore, ZA is considered a mainstay of treatment for bone metastases secondary to solid tumors such as breast cancer [21]. As an adjuvant therapy, ZA was also shown to prevent bone metastases secondary to the last-mentioned cancer type [22]. In addition, ZA has been used for other cancers such as lung, kidney and prostate [13]. Systematic reviews and network *meta*-analyses (NMAs) on bone metastases concluded that ZA was associated with a statistically significant decrease in SREs when compared to placebo [23], [24]. However, no NMA was performed regarding other clinical parameters such as overall survival, progression-free survival or level of pain [23], [24].

The purpose of this systematic review and Bayesian NMA is to compare ZA to other treatment options in its ability to improve overall survival, decrease the incidence of skeletal-related events and reduce pain in patients with bone metastases secondary to any primary tumors.

## 2 Materials and Methods

### 2.1 Search strategy

A systematic review was performed according to the Preferred Reporting Items for Systematic Reviews and Meta-Analyses (PRISMA) guidelines [25]. The search was performed in the following databases: PubMed, Embase (via Ovid) and Web of Science. All databases were searched from inception to May 5th, 2022. The following keywords were used: solid tumor, lung neoplasm, kidney neoplasm, breast neoplasm, prostate neoplasm, ZA and bone metastasis. Synonyms of these terms were also used but not listed here (Supplementary Table 1–3). Each reference section was hand screened to identify additional studies. Endnote was used to remove the duplicates and tract removal or addition of studies.

### 2.2 Eligibility criteria

#### 2.2.1 Population

Studies of human adults with bone metastases secondary to any solid tumors, regardless of the language, age or sex, were included.

#### 2.2.2 Interventions and comparator

Randomized controlled trials and non-randomized quasi-experimental studies of systemic ZA administration for patients with bone metastases and any comparator, including placebo, were included. Subgroup analyses of broader studies that were already included in our study were excluded.

#### 2.2.3 Outcomes

The primary outcomes are 1) the development of a new skeletal-related event, defined as new bone metastases, pathological fractures, spinal cord compression or disabling pain; 2) time to developing a first SRE during the study; 3) disease progression-free survival (PFS); and 4) overall survival (OS). The secondary outcome is pain at 3, 6 and 12 months after treatment as measured by the brief pain inventory (BPI) or a visual analogue score (VAS). Data from both scores were pooled since they share the same psychometric dimensions.

### 2.3 Screening and abstraction process

Titles and abstracts of articles identified were screened independently by two researchers (JPL and FGB). Any citation identified by either investigator as potentially relevant was then advanced to full-text review. Any discrepancies were resolved through a consensus discussion with a senior member of the research team (EA or JRG). Once full texts had been included in the study, data abstraction was performed in pairs (JPL, FGB, or JRG).

Treatments were categorized as ZA 4 or 8 mg every 3–4 weeks. When combined with any other drug (i.e. chemotherapeutics), regardless of ZA dosage, ZA was categorized as ZA in combination. Identified comparators included androgen blockade drugs, everolimus, denosumab, docetaxel, other bisphosphonates, and strontium-89 (Sr-89). Primary cancer type was categorized as breast, lung, prostate or other. The time of ZA treatment was categorized as before, during or after chemotherapy treatment.

For the relative effectiveness outcomes, the data extracted was the number of patients who presented with a SRE; its odds ratio (OR) and its 95 % credible intervals (CrI). Regarding the time to a first on-study SRE, OS, and PFS, hazard ratios (HR) and 95 % credible intervals (CrI) were calculated. Finally, for pain, the mean and standard error of the mean of the treatment effect were extracted and converted into standard mean differences (SMD). For HR calculation where data was not explicitly stated, Kaplan-Meier survival curves were used with the method described by Tierney et al. [26] using the Engauge Digitizer software, version 10.11 to estimate the values.

### 2.4 Risk of bias

Risk of bias in individual studies was assessed using the Risk of Bias v.2 tool [27]. Assessments were done in duplicate (JPL and FBG supervised by EA). If present, discrepancies were resolved between authors and if necessary, with other members of the research team (JRG and EA). Data visualization was done using the Robvis tool [28].

### 2.5 Calculations and statistical analysis

Following the guide by Harrer et al. [29], a NMA was performed under a Bayesian framework for each outcome using a random-effects model with the *gemtc* and *rjags* packages in the R v.4.1.0 [30]. For the binary outcome of SREs development, the model corresponded to a generalized linear model with a logit link. For time to event outcomes (time to develop a first SRE, OS and PFS), the model corresponded to a generalized linear model with a c-loglog link. Finally, for the continuous outcomes of pain, the model corresponded to a generalized linear model with an identity link. The between-study heterogeneity was assumed to be constant. Non-informative prior distributions was used for effectiveness model parameters given current uncertainty of the relative effectiveness of the treatments. Convergence of the 4 chains using the Gelman-Rubin statistic was used to assess convergence of the algorithm for the prior distributions, as well as visual inspection of trace plots. Convergence was deemed achieved if the potential scale reduction factor was lower than 1.05. Consistency of the network model was assessed through the nodesplit method. However, because the geometry of the networks had paucity of head-to-head trials, nodesplits were achieved only for some treatments.

A network plots was generated for each analysis. Summary results were presented as either OR, HR, or SMD with 95 % CrI. Treatment rankings were summarized by the surface under the cumulative ranking (SUCRA) that expressed the probability (0.00–1.00) of effectiveness. Each treatment has been compared with an ideal treatment ranked always first without uncertainty. Meta-regression analyses were done to assess whether the type of primary cancer or time of treatment with respect to chemotherapy administration influenced the primary outcomes.

## 3 Results

**Fig. 1:**
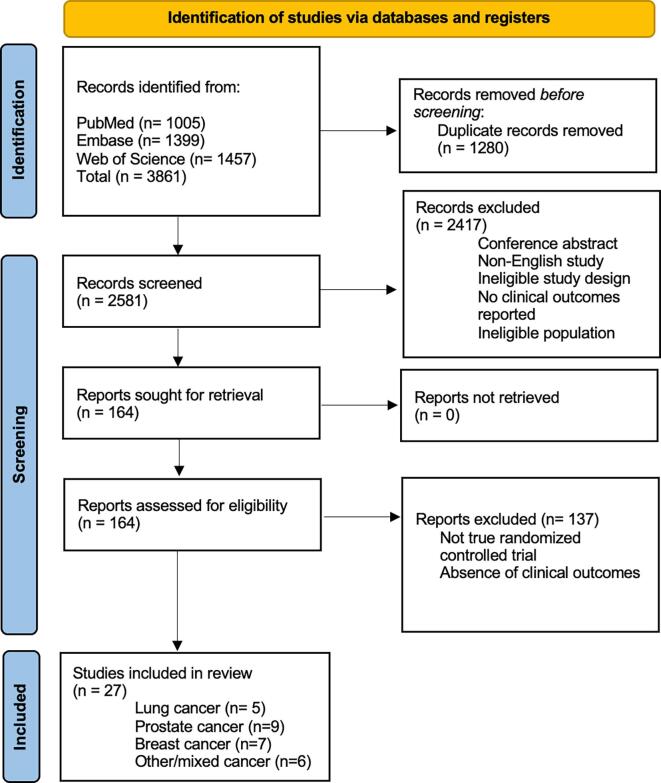
PRISMA figure illustrating the inclusion of the studies with the steps involved in the screening process. The separation highlighted in blue on the left shows the different sections of the process. (For interpretation of the references to colour in this figure legend, the reader is referred to the web version of this article.)

**Supplementary figure 1:**
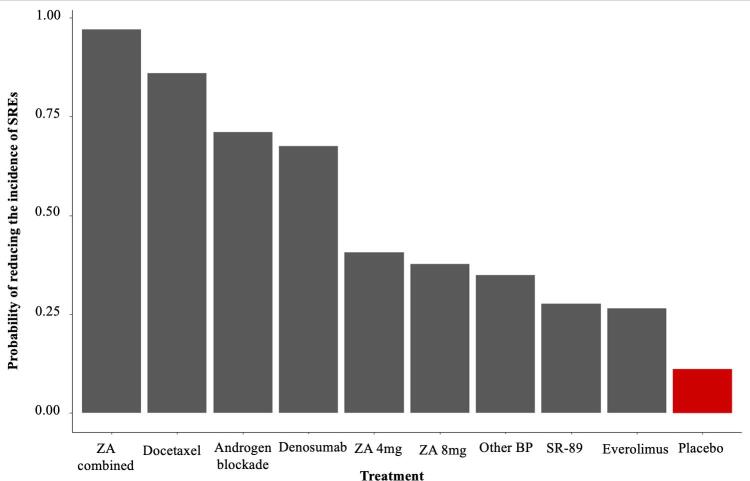


**Supplementary figure 2:**
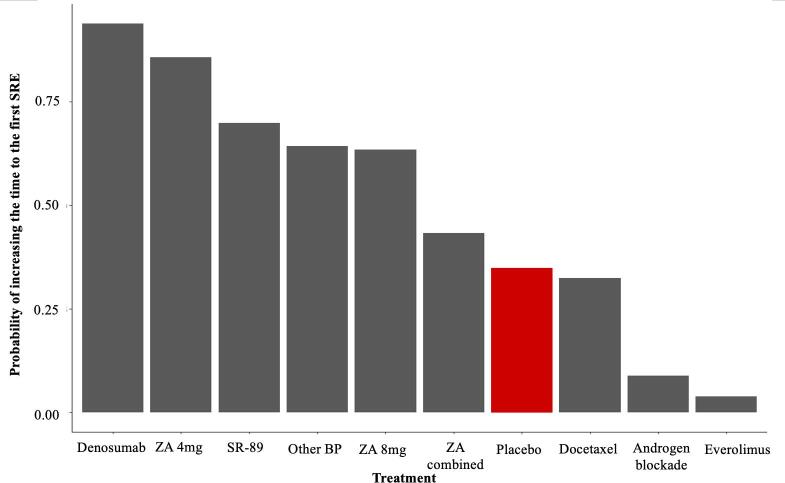


**Supplementary figure 3:**
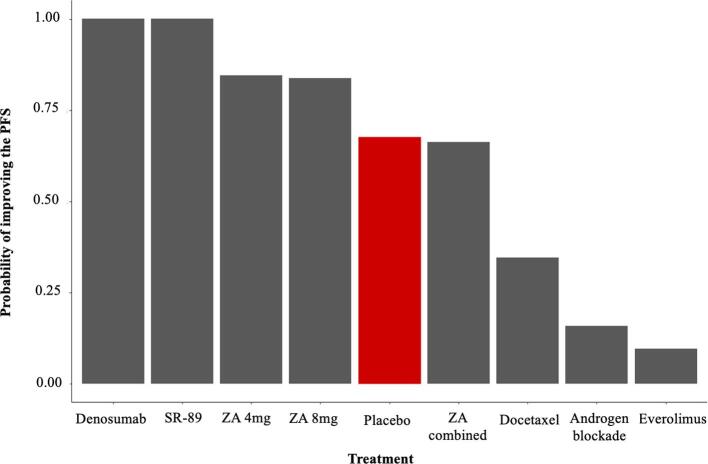


**Supplementary figure 4:**
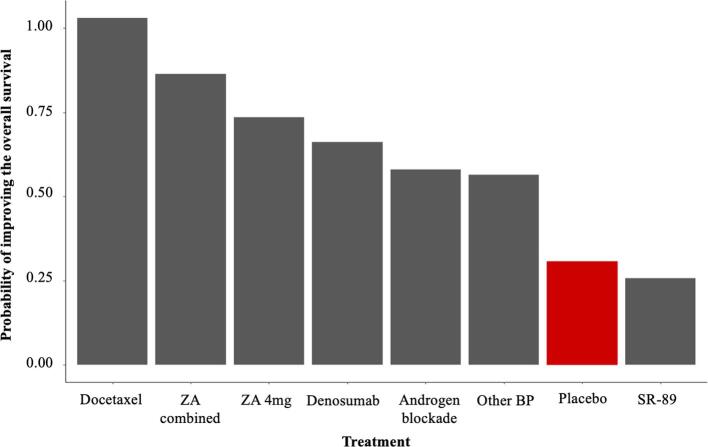


**Supplementary figure 5:**
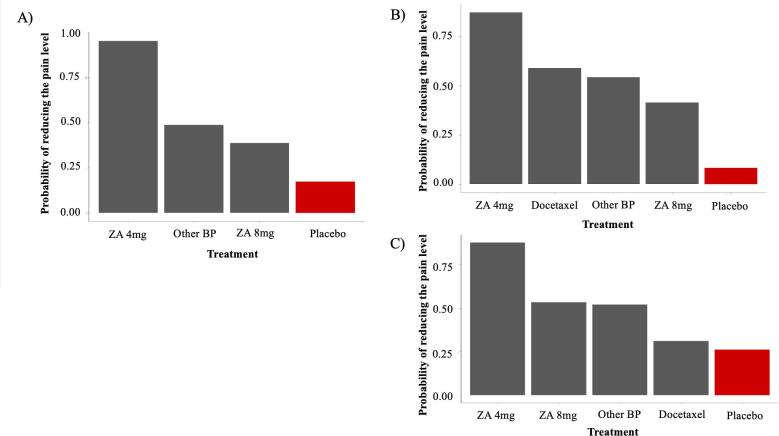


Our search yielded 3861 titles (Fig. 1). After removing duplicates, 2581 articles were screened based on titles and abstracts. After reviewing the bibliography of each paper, one more article was found to be eligible for the NMA. A total of 27 articles remained relevant based on the inclusion and exclusion criteria. The studies were divided based on the location of the primary tumors: lung (5), prostate (9), breast (7) and other/mixed (6). A total of 19,824 patients and 7 therapies were compared to ZA. The most frequent comparator was placebo. The mean number of participants in each group was 333 (range 15–1026) (Table 1). For each outcome, separation based on primary cancer or by time of administration was not associated with reduced heterogeneity of the data (Supplementary Table 4). The SUCRA was calculated for each outcome based on the different treatments. Cumulative rankings are represented in Supplementary Figs. 1–5. In addition, separation of prostate cancer studies based on castration resistant, castration sensitive or hormone naïve was performed. However, no differences were found in these three subcategories. This most likely represents underpower of the analysis as only 4, 2 and 2 studies were included for each category, respectively.

**Table 1:** List of included studies. The data of each randomized controlled trial is represented in the columns. NSCLC = Non-small cell lung cancer, ZA = Zoledronic Acid, SR-89 = Strontium-89, SC = Subcutaneous, IV = Intravenous, MBq = Megabecquerel.

**Authors**	**Year**	**Primary cancer**	**Time of treatment compared to the main therapy**	**Treatment**	**Population size for treatment (n)**	**Comparator**	**Population size for comparator (n)**
Barrett-Lee, P[33]	2014	Breast cancer	During	ZA- 4 mg/ 4 weeks	699	Ibandronic acid- 50 mg daily	705
Broom, R. J [34]	2015	Renal cell carcinoma	Before	Everolimus and ZA - 4 mg/ 4 weeks	15	Everolimus- 10 mg daily	15
Choudhury, K. B [31]	2011	NSCLC + others	During	ZA − 4 mg/ 4 weeks	60	1. Ibandronate- 6 mg/ 4 weeks 2. Pamidronate- 90 mg/ 4 weeks	1. 65 2. 62
Cleeland, C. S [76]	2013	Breast cancer	Before	ZA − 4mg/ 4 weeks + SC placebo	1020	Denosumab- 120 mg/ 4 weeks + IV placebo	1026
Fizazi, K [35]	2011	Prostate cancer	After	ZA − 4 mg/ 4 weeks + SC placebo	951	Denosumab- 120 mg/ 4 weeks + IV placebo	950
Francini, F [36]	2011	NSCLC	During	ZA − 4mg/ 4 weeks	28	Ibandronic acid- 50 mg/day	27
Henry, D [37]	2014	NSCLC	Before	ZA - 4 mg/ 4 weeks + SC placebo	797	Denosumab SC- 120 mg/ 4 weeks with placebo IV	800
Hilton, J. F [38]	2018	Breast cancer	After	ZA − 4 mg/ 4 weeks	38	Pamidronate	35
Jacobs, C [39]	2016	Breast cancer	During	ZA − 4 mg/ 4 weeks	35	Pamidronate	38
James, N [40]	2016	Prostate cancer	Before	Docetaxel, ZA − 4 mg/ 4 weeks	188	1. Docetaxel- 75 mg/m2/ 3 weeks, 2. Docetaxel and ZA 3. Docetaxel + Sr-89.	1. 191 2. 188 3. 190
Kamba, T [41]	2016	Prostate cancer	Before	ZA − 4 mg/ 4 weeks with combined androgen blockade	115	80 mg of bicalutamide orally once a day and subcutaneous luteinizing hormone–releasing hormone agonist every 4 or 12 weeks	112
Kohno, N [42]	2005	Breast cancer	During	ZA − 4 mg/ 4 weeks	114	Placebo	114
Martin, M [43]	2012	Breast cancer	Before	ZA − 4 mg/ 4 weeks + SC placebo	1020	Denosumab- 120 mg/ 4 weeks + IV placebo	1026
Murakami, H [44]	2014	NSCLC	After	ZA − 4 mg/ 4 weeks with Docetaxel	50	Docetaxel- 60 mg/m2	50
Pan, Y [45]	2014	Prostate cancer	After	Docetaxel-based chemotherapy + ZA - 4 mg/ 3 weeks	53	Docetaxel-based chemotherapy- 75 mg/m2 of docetaxel for 21 days and placebo	52
Pandya, K. J [56]	2010	NSCLC	After	Docetaxel, Carboplatin and ZA − 4 mg/ 4 weeks	64	Docetaxel- 75 mg/m2 for 1 h and carboplatin- IV for 15 min	64
Price, N [32]	1999	NSCLC + others	Before	ZA - A. 4 mg / 3 weeks B. 8 mg/ 3 weeks; reduced to 4 mg/ 3 weeks	A. 254 B. 265	Placebo	247
Rosen, L. S [46]	2003	Multiple myeloma and breast cancer	During	ZA - A. 4 mg/ 4 weeks B. 8 mg/ 4 weeks; later switched to 4 mg/ 4 weeks	A. 564 B. 526	Pamidronate- 90 mg/ 3 or 4 weeks	558
Rosen, L. S [47]	2004	NSCLC + others	During	ZA - A. 4 mg/ 4 weeks B. 8 mg/ 4 weeks; later switched to 4 mg/ 4 weeks	A. 257 B. 266	Placebo	250
Saad, F [48]	2002	Prostate cancer	During	ZA - A. 4 mg / 4 weeks B. 8 mg/ 4 weeks;later switched to 4 mg/ 4 weeks	A. 214 B. 221	Placebo	208
Smith, M. R [50]	2014	Prostate cancer	Before	ZA − 4 mg/ 4 weeks	323	Placebo	322
Smith, M. R. [49]	2015	Prostate cancer	After	ZA − 4 mg/ 4 weeks	951	Denosumab- 120 mg/ 4 weeks	950
Stopeck, A.T [51]	2010	Breast cancer	During	ZA − 4 mg/ 4 weeks + SC placebo	1020	Denosumab- 120 mg/ 4 weeks + IV placebo	1026
Ueno, S [52]	2013	Prostate cancer	Before	ZA − 4 mg/ 4 weeks with combined androgen blockade	29	Combined androgen blockade − 80 mg/ day	31
Wang, F [53]	2013	Prostate cancer	Before	ZA − 4 mg/ 4 weeks	69	Clodronate- 1600 mg / day	68
Wang, Y [55]	2013	NSCLC	Before	Sr-89, 0.9 % sodium chloride and ZA − 4 mg/ 4 weeks	45	1. ZA − 4 mg every 3/4 weeks 2. Sr-89–150 MBq of Sr-89 every 6 months 3. Chemotherapy	1. 45 2. 45 3. 45
Zaghloul, MS [54]	2010	Bladder cancer	After	ZA − 4 mg/ 4 weeks	20	Placebo	20

### 3.1 Study quality

Among the included studies, 14 had an overall risk of bias considered “low risk”, 11 studies had “some concerns” and 2 were classified “high risk”. These two studies, one from Choudhoury et al. [31] and another one from Price et al. [32] had biases due to deviation from intended intervention. Furthermore, Price et al. [32] had high risk bias due to missing outcome data (Supplementary Fig. 6).

### 3.2 Skeletal-related events

**Fig. 2:**
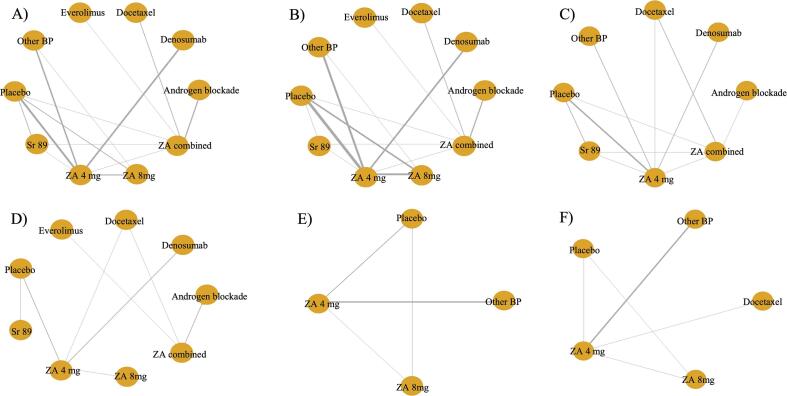
**Network graphs for each different outcome analyzed in the study.** The orange nodes correspond to each different study. The thickness of the grey lines is proportional to the number of trials between each pair of treatment nodes. More precisely, there is A) Time to first skeletal-related event, B) number of skeletal-related events, C) Overall survival, D) progression-free survival, E) pain at 3 months and F) Pain at 6- and 12-month. ZA = Zoledronic Acid, BP = Bisphosphonate, SR-89 = Strontium-89. (For interpretation of the references to colour in this figure legend, the reader is referred to the web version of this article.)

**Fig. 3:**
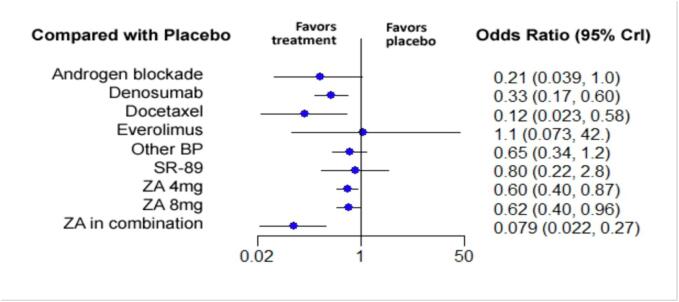
**Forest plot of the impact of the different therapies in preventing the development of a SRE when compared to placebo.** On the left, the different therapies are labelled. On the right, the odds ration corresponding to each therapy when compared to placebo are represented. In parenthesis, the 95 % credible intervals are shown. BP = Bisphosphonate, ZA = Zoledronic Acid, SR-89 = Strontium-89, CrI = Credible Intervals.

A total of 24 studies were included in the analysis of the SREs [32], [33], [34], [35], [36], [37], [38], [39], [40], [41], [42], [43], [44], [45], [46], [47], [48], [49], [50], [51], [52], [53], [54], [55]. Nine therapies were compared together and with placebo. These include ZA 4 mg, ZA 8 mg, ZA combined with another treatment, androgen blockade, denosumab, docetaxel, everolimus, Sr-89 and all the other bisphosphonates pooled in one group (Fig. 2). The NMA showed that three therapies were associated with a clinically significant decrease in the number of SREs when compared to placebo. Among them, ZA in combination with chemotherapy or hormone therapy was superior to placebo for preventing SREs with an OR of 0.079 (95 % CrI:0.022–0.27), followed by docetaxel and denosumab with ORs of 0.12 (95 % CrI:0.023–0.58) and 0.33 (95 % CrI:0.17–0.60) respectively. The relative impact of the different treatment groups when compared to placebo is depicted in Fig. 3. Pairwise comparison between the different treatments is depicted in the league table (Supplementary Table 5).

### 3.3 Time to a first on-study skeletal-related event

**Fig. 4:**
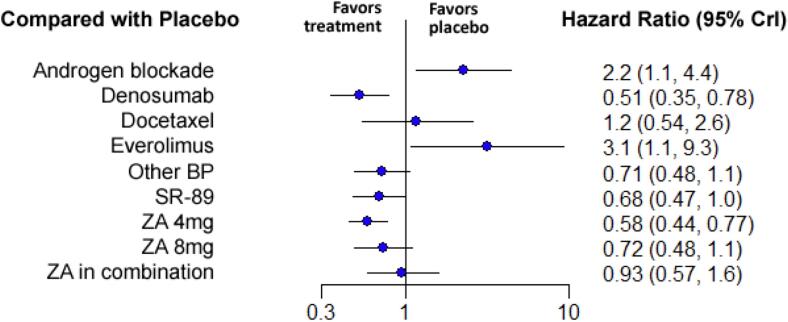
**Forest plot of the relative effectiveness of the different therapies to modify the time to develop a first SRE when compared to placebo.** On the left, the different therapies are labelled. On the right, the hazard ratios corresponding to each therapy when compared to placebo are represented. In parenthesis, the 95 % credible intervals are shown. BP = Bisphosphonate, ZA = Zoledronic Acid, SR-89 = Strontium-89, CrI = Credible Intervals.

Twenty studies with 10 different treatments were included in the analysis of the time to develop a first on-study SRE [33], [34], [35], [37], [38], [40], [41], [43], [44], [45], [46], [47], [48], [49], [50], [51], [52], [53], [54], [55]. Included therapies were placebo, ZA 4 mg, ZA 8 mg, ZA combined with another treatment, androgen blockade, denosumab, docetaxel, everolimus, Sr-89 and all other bisphosphonates grouped (Fig. 2). When compared to placebo, the treatment with the greatest relative effectiveness for protecting against the development of a first on-study SRE was denosumab (HR 0.51 with 95 % CrI:0.35–0.78), followed by ZA 4 mg (HR 0.58 with 95 % CrI:0.48–0.77). No other treatment had a statistically significant increase in the time to treat a first SRE. The relative effectiveness of the different therapies when compared to placebo is depicted in Fig. 4. Pairwise comparison between the different treatments is depicted in the league table (Supplementary Table 6).

### 3.4 Progression-free survival

**Fig. 5:**
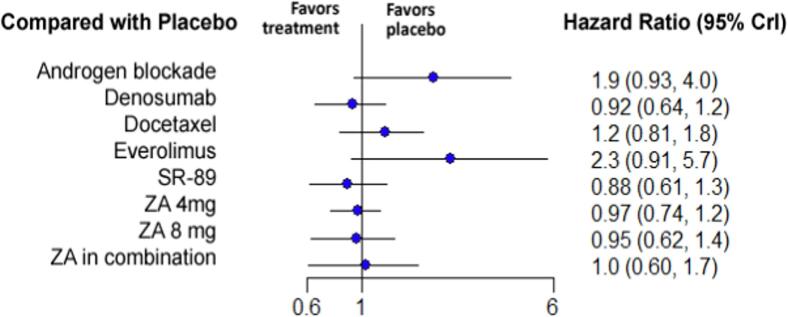
**Forest plot of the relative effectiveness of the different therapies for progression-free survival when compared to placebo.** On the left, the different therapies are labelled. On the right, the hazard ratios corresponding to each therapy when compared to placebo are represented. In parenthesis, the 95 % credible intervals are shown. ZA = Zoledronic Acid, SR-89 = Strontium-89, CrI = Credible Intervals.

A total of 10 studies were included in the analysis of the progression-free survival [34], [35], [37], [40], [41], [44], [46], [50], [52], [56]. Nine different therapies were compared including placebo, ZA 4 mg, ZA 8 mg, ZA combined with another treatment, androgen blockade, denosumab, docetaxel, everolimus and strontium-89 (Fig. 2). The treatment with the highest relative effectiveness was Sr-89 (HR 0.88 with 95 % CrI:0.61–1.3), followed by denosumab (HR 0.92 with 95 % CrI:0.64–1.2) and ZA 8 mg (HR 0.95 with 95 % CrI:0.62–1.4). However, no treatment was statistically significant for increasing the PFS. The relative effectiveness of the different therapies is depicted in Fig. 5. Pairwise comparison between the different treatments is depicted in the league table (Supplementary Table 7).

### 3.5 Overall survival

**Fig. 6:**
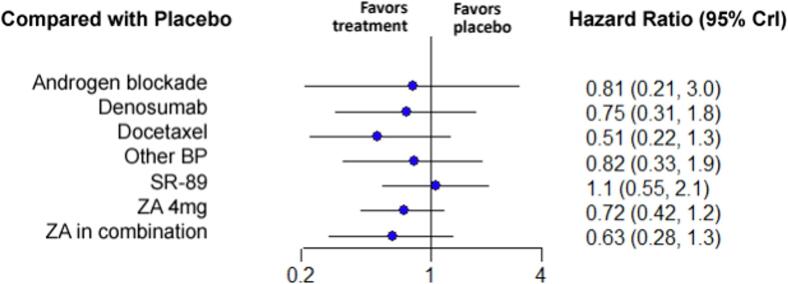
**Forest plot of the relative effectiveness of the different therapies for overall survival when compared to placebo.** On the left, the different therapies are labelled. On the right, the hazard ratios corresponding to each therapy when compared to placebo are represented. In parenthesis, the 95 % credible intervals are shown. BP = Bisphosphonate, ZA = Zoledronic Acid, SR-89 = Strontium-89, CrI = Credible Intervals .

For the overall survival, 12 studies with 8 treatments were included in the analysis [33], [35], [40], [41], [44], [45], [50], [51], [53], [54], [55], [56]. Included therapies were placebo, ZA 4 mg, ZA combined with another treatment, androgen blockade, denosumab, docetaxel, Sr-89 and all other bisphosphonates pooled (Fig. 2). The treatment associated with the highest survival was docetaxel (HR 0.51 with 95 % CrI:0.22–1.3), followed by ZA in combination with chemotherapy or hormone therapy (HR 0.63 with 95 % CrI:0.28–1.3) and ZA 4 mg (HR 0.72 with 95 % CrI:0.42–1.2). However, no treatment was associated with a statistically significant decrease in overall survival when compared with placebo. The relative effectiveness of the different therapies when compared to placebo is illustrated in Fig. 6. Pairwise comparison between the different treatments is depicted in the league table (Supplementary Table 8).

### 3.6 Pain

**Fig. 7:**
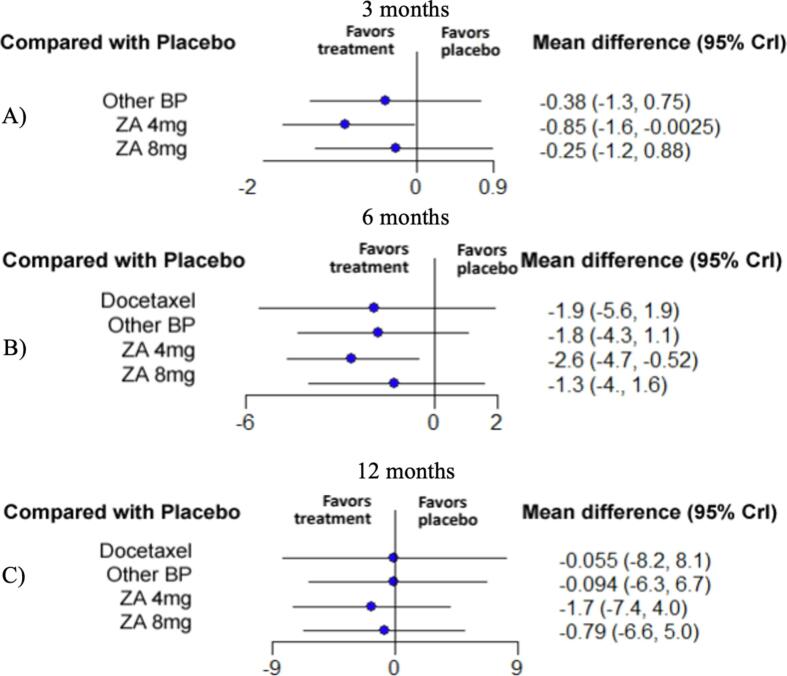
Forest plot of the relative effectiveness of the different therapies to modify pain BPI/VAS scores when compared to placebo at A) 3 months, B) 6 months and C) 12 months. On the left, the different therapies are labelled. On the right, the standardized mean difference corresponding to each therapy when compared to placebo is represented. In parenthesis, the 95 % credible intervals are shown. BP = Bisphosphonate, ZA = Zoledronic Acid, CrI = Credible Intervals.

A total of 6 studies were included in the analysis of the pain at the 3- and 6-month follow-ups, with four and five different treatments, respectively [31], [33], [45], [48], [53], [54]. Five studies with 5 treatments were selected for the 12-month comparison [31], [33], [45], [48], [53]. These treatments include ZA 4 mg, ZA 8 mg, docetaxel and all the other bisphosphonates in one group (Fig. 2). At 3 and 6 months, ZA 4 mg was significantly superior to placebo for reducing pain with a SMD of −0.85 (95 % CrI:-1.6, −0.0025) and −2.6 (95 % CrI:-4.7, −0.52) respectively. No other treatment was statistically superior to placebo at 3 and 6 months. At 12 months, no treatment was statistically superior to placebo (Fig. 7). Pairwise comparison between the different treatments is depicted in the league table (Supplementary Table 9).

## 4 Discussion

Zoledronic acid has been shown to improve the quality of life of patients with bone metastases while maintaining an acceptable safety profile for long-term use [57], [58]. Our Bayesian NMA showed that ZA decreases the number of SREs, increases the time to a first on-study SRE and reduces short-term pain level in patients with metastatic bone tumors.

Only few systematic reviews and *meta*-analyses were published on the use of ZA in patients with bone metastases. When compared to ZA, denosumab was significantly better at delaying the time to the first skeletal-event and reducing pain. Overall survival and disease progression were similar in both groups [59], [60]. These results are in accordance with our study regarding the time to the first skeletal-related event. However, our study demonstrated superiority of ZA 4 mg in reducing pain at the 3- and 12-month follow ups given the paucity of data for the denosumab group. A NMA comparing the different BPs with denosumab concluded that denosumab was superior to ZA in reducing the number of SREs [23]. This is coherent with our results which showed more benefits with denosumab than ZA 4 mg alone. However, the current *meta*-analysis demonstrates that ZA, when combined with chemotherapy or hormone therapy, is superior to denosumab alone.

While clinical trials include data that is highly curated, the use of ZA for breast cancer and bone metastases has been proven to be efficacious in real-world evidence [61]. Futhermore, a cost-effectiveness analysis has shown that bisphosphonates, compared to no therapy, are either cost-saving or highly cost effective [62]. In the same study, ZA was shown to be the most effective and less expensive of all options for reducing SREs and improving the quality of life of patients with bone metastases [62]. Another cost-analysis study for the same type of tumors demonstrated that the cost of generic ZA treatment every 3 months was approximately ninefold lower than monthly denosumab therapy in addition to being more cost-effective in reducing the number of SREs [63]. Altogether, this supports our findings that ZA is the best option for preventing SREs secondary to bone metastases.

Nevertheless, ZA treatment has some limitations such as osteonecrosis of the jaw. This complication was shown to be higher in smokers and in patients with poor dental hygiene [57], [64]. Patients also reported other less serious symptoms including flu-like symptoms, diarrhea, nausea and heartburn [57]. A more significant clinical limitation is the use of ZA in patients with pre-existing renal dysfunctions since ZA is predominantly excreted through the kidney and therefore is associated with a risk of nephrotoxicity [65], [66].

Based on the FDA recommendations [67], the ideal ZA dose is 4 mg infused over no less than 15 min every 3 to 4 weeks, which results in a peak serum concentration of 1–3 µM for a few hours following systemic administration [68]. Local continuous administration of drugs is an emerging approach [69], [70]. This would ensure high drug concentrations at the site and reduce the systemic absorption [70], [71]. Therefore, local delivery of ZA would reduce the risk of experiencing side effects [57], [72]. This can be achieved by using impregnated 3D-printed nanoporous scaffolds [73]. In vitro studies have shown that with a 3D-printer, it is possible to customize a model and insert it in a bone defect to allow bone marrow stem cells to infiltrate, adhere, proliferate, and form new bone [73]. Such approach showed promising results where approximately 3 µM of ZA impregnated into nanoscaffolds or beads was enough to achieve the same therapeutic concentration locally as the systemic dose of 4 mg upon intravenous administration [70], [71]. As shown in Table 1, every study involving a ZA infusion of 8 mg amended its protocol to decrease the infusion to 4 mg [32], [46], [47]. This is likely secondary to the detrimental effect of a higher systemic dosage of ZA. In fact, both studies by Rosen et al. reported an increase in creatinine level with 8 mg infusion [46], [47]. This effect might explain why ZA 4 mg performed better than ZA 8 mg.

Another area of research focuses on the combination of ZA with other treatments [74], [75]. A synergistic action would allow the patients to receive a lower dose from both medications, therefore reducing the risk of side effects. Investigations demonstrated that ZA combined with chemotherapy had a synergistic effect at the tumor site in vitro [74]. A similar synergistic effect was found in a study conducted with mice where immunotherapy and ZA had an increased anti-tumor efficacy [75].

An important strength of our study is its design. The network *meta*-analysis allows for a broader comparison of articles compared to a regular *meta*-analysis. We were able to analyze every treatment found in the included studies and compare them with each other. On the other hand, this study is limited by the reported data of the included studies. While primary cancer and time of administration were not associated with reduced heterogeneity, it was not possible to control for other factors given the lack of data. Future studies should attempt to control for other parameters such as Charlson comorbidity index and extent of disease.

## 5 Conclusion

Our Bayesian NMA shows the benefits of ZA 4 mg in reducing the incidence of new SREs, increasing the time to the development of a SRE, and decreasing pain in the short term. ZA in combination with chemotherapy or hormone therapy had the highest relative effectiveness at reducing the incidence of SREs. However, ZA 4 mg alone also showed a statistically significant improvement in the time to a first on-study SRE when compared to placebo. Unfortunately, for the overall survival and progression free survival, no treatment was associated with a statistically significant increase when compared with placebo.

## CRediT authorship contribution statement

**Justin-Pierre Lorange:** Investigation, Data curation, Writing - original draft. **Jose Ramirez Garcia Luna:** Software, Formal analysis, Writing – review & editing. **Frédéric Grou-Boileau:** Investigation. **Derek Rosenzweig:** Writing – review & editing. **Michael H. Weber:** Writing – review & editing, Supervision. **Elie Akoury:** Conceptualization, Supervision, Project administration, Methodology, Writing - review & editing.

## Declaration of Competing Interest

The authors declare that they have no known competing financial interests or personal relationships that could have appeared to influence the work reported in this paper.

This research was funded by AO Start-Up grant number S-16-138W to M.H.W. and the Cancer Research Society (CRS) grant number 23014 to M.H.W. and D.H.R., by internal start-up funding from the Research Institute of the McGill University Health Centre (RI-MUHC) to D.H.R. and the Réseau de Recherche en Santé Buccodentaire et Osseuse (RSBO) to M.H.W. and D.H.R. E.A. was supported by postdoctoral fellowships from the RI-MUHC, RSBO and from McGill University-Faculty of Medicine.

## Figures and Tables

**Supplementary figure 6:**