# Supplementary material for: Management of bone metastasis with zoledronic acid: A systematic review and Bayesian network meta-analysis
Source: J Bone Oncol. 2023 Feb 9;39:100470. doi: 10.1016/j.jbo.2023.100470 (PMC9969300; doi:10.1016/j.jbo.2023.100470)
Supplement: Supplementary data 1 [file mmc1.docx]

**Supplementary Table 1.** Terms used for the search in PubMed.

| Search # | Search details | Results |
| --- | --- | --- |
| 1 | "solid tumor"[All Fields] | 45,346 |
| 2 | "solid tumors"[All Fields] | 14,957 |
| 3 | "Lung Neoplasm"[All Fields] | 262,687 |
| 4 | "Lung Neoplasms"[MeSH Terms] | 575 |
| 5 | "Lung cancer"[All Fields] | 190,707 |
| 6 | "Kidney Neoplasms"[MeSH Terms] | 81,538 |
| 7 | "renal cancer"[All Fields] | 7,093 |
| 8 | "Kidney neoplasm"[All Fields] | 179 |
| 9 | "renal neoplasm"[All Fields] | 653 |
| 10 | "Breast Neoplasms"[MeSH Terms] | 328,793 |
| 11 | "breast cancer"[All Fields] | 322,752 |
| 12 | "breast neoplasm"[All Fields] | 1,060 |
| 13 | "prostatic neoplasms"[MeSH Terms] | 143,321 |
| 14 | "prostate cancer"[All Fields] | 135,431 |
| 15 | "prostate neoplasm"[All Fields] | 176 |
| 16 | "Zoledronate"[All Fields] | 1,521 |
| 17 | "Zoledronic Acid"[MeSH Terms] | 3,864 |
| 18 | "Zoledronic Acid"[All Fields] | 5,474 |
| 19 | "bone metastasis"[All Fields] | 8,960 |
| 20 | "bone metastases"[All Fields] | 13,199 |
| 21 | "solid tumors"[All Fields] OR "solid tumor"[All Fields] | 57,111 |
| 22 | "Lung Neoplasms"[MeSH Terms] OR "Lung Neoplasm"[All Fields] OR "Lung cancer"[All Fields] | 322,690 |
| 23 | "Kidney Neoplasms"[MeSH Terms] OR "renal cancer"[All Fields] OR "Kidney neoplasm"[All Fields] OR "renal neoplasm"[All Fields] | 84,177 |
| 24 | "Breast Neoplasms"[MeSH Terms] OR "breast cancer"[All Fields] OR "breast neoplasm"[All Fields] | 423,588 |
| 25 | "prostatic neoplasms"[MeSH Terms] OR "prostate cancer"[All Fields] OR "prostate neoplasm"[All Fields] | 178,436 |
| 26 | "solid tumors"[All Fields] OR "solid tumor"[All Fields] OR "Lung Neoplasms"[MeSH Terms] OR "Lung Neoplasm"[All Fields] OR "Lung cancer"[All Fields] OR "Kidney Neoplasms"[MeSH Terms] OR "renal cancer"[All Fields] OR "Kidney neoplasm"[All Fields] OR "renal neoplasm"[All Fields] OR "Breast Neoplasms"[MeSH Terms] OR "breast cancer"[All Fields] OR "breast neoplasm"[All Fields] OR "prostatic neoplasms"[MeSH Terms] OR "prostate cancer"[All Fields] OR "prostate neoplasm"[All Fields] | 1,008,977 |
| 27 | "Zoledronate"[All Fields] OR "Zoledronic Acid"[MeSH Terms] OR "Zoledronic Acid"[All Fields] | 6,079 |
| 28 | "bone metastasis"[All Fields] OR "bone metastases"[All Fields] | 19,522 |
| 29 | ("solid tumors"[All Fields] OR "solid tumor"[All Fields] OR ("Lung Neoplasms"[MeSH Terms] OR "Lung Neoplasm"[All Fields] OR "Lung cancer"[All Fields]) OR ("Kidney Neoplasms"[MeSH Terms] OR "renal cancer"[All Fields] OR "Kidney neoplasm"[All Fields] OR "renal neoplasm"[All Fields]) OR ("Breast Neoplasms"[MeSH Terms] OR "breast cancer"[All Fields] OR "breast neoplasm"[All Fields]) OR ("prostatic neoplasms"[MeSH Terms] OR "prostate cancer"[All Fields] OR "prostate neoplasm"[All Fields])) AND ("Zoledronate"[All Fields] OR "Zoledronic Acid"[MeSH Terms] OR "Zoledronic Acid"[All Fields]) AND ("bone metastasis"[All Fields] OR "bone metastases"[All Fields]) | 1,005 |

**Supplementary Table 2.** Terms used for the search in Embase

| Search # | Search details | Results |
| --- | --- | --- |
| 1 | solid tumors.mp. or solid malignant neoplasm/ | 70297 |
| 2 | solid tumor.mp. | 54857 |
| 3 | 1 or 2 | 99033 |
| 4 | lung neoplasms.mp. or lung tumor/ | 79673 |
| 5 | lung neoplasm.mp. | 1146 |
| 6 | lung cancer.mp. or lung cancer/ | 307694 |
| 7 | lung cancers.mp. | 17691 |
| 8 | 4 or 5 or 6 or 7 | 359253 |
| 9 | kidney neoplasms.mp. or kidney tumor/ | 40421 |
| 10 | kidney neoplasm.mp. | 330 |
| 11 | renal cancer.mp. or kidney cancer/ | 25899 |
| 12 | renal neoplasms.mp. or kidney carcinoma/ | 60933 |
| 13 | renal neoplasm.mp. | 919 |
| 14 | kidney cancer.mp. | 24647 |
| 15 | 9 or 10 or 11 or 12 or 13 or 14 | 111771 |
| 16 | breast neoplasms.mp. or breast tumor/ | 104449 |
| 17 | breast neoplasm.mp. | 1904 |
| 18 | breast cancer.mp. or breast cancer/ | 508844 |
| 19 | 16 or 17 or 18 | 555105 |
| 20 | prostatic neoplasms.mp. or prostate tumor/ | 38332 |
| 21 | prostate cancer.mp. or prostate cancer/ | 226626 |
| 22 | prostate neoplasms.mp. | 1363 |
| 23 | prostate neoplasm.mp. | 425 |
| 24 | 20 or 21 or 22 or 23 | 242379 |
| 25 | 3 or 8 or 15 or 19 or 24 | 1238534 |
| 26 | Zoledronic acid.mp. or zoledronic acid/ | 16340 |
| 27 | Zoledronate.mp. | 2026 |
| 28 | 25 or 26 | 16483 |
| 29 | bone metastasis.mp. or bone metastasis/ | 48097 |
| 30 | bone metastases.mp. | 19573 |
| 31 | bone mets.mp. | 249 |
| 32 | 27 or 28 or 29 | 51668 |
| 33 | 30 and 31 and 32 | 1399 |

**Supplementary Table 3.** Terms used for the search in Web of Science

| Search # | Search details | Results |
| --- | --- | --- |
| 1 | TOPIC: ("solid tumors") | 51,984 |
| 2 | TOPIC: ("solid tumor") | 11807 |
| 3 | TOPIC: ("lung neoplasms") | 3,071 |
| 4 | TOPIC: ("lung neoplasm") | 775 |
| 5 | TOPIC: ("lung cancer") | 256,215 |
| 6 | TOPIC: ("lung cancers") | 11313 |
| 7 | TOPIC: ("kidney neoplasms") | 1,528 |
| 8 | TOPIC: ("kidney neoplasm") | 268 |
| 9 | TOPIC: ("renal cancer") | 6,535 |
| 10 | TOPIC: ("renal neoplasms") | 963 |
| 11 | TOPIC: ("renal neoplasm") | 564 |
| 12 | TOPIC: ("kidney cancer") | [5,982](https://apps-webofknowledge-com.proxy3.library.mcgill.ca/summary.do?product=WOS&doc=1&qid=12&SID=5Dr8CsPeKmUnMsPCPCt&search_mode=GeneralSearch&update_back2search_link_param=yes) |
| 13 | TOPIC: ("breast neoplasms") | 7,144 |
| 14 | TOPIC: ("breast neoplasm") | 1,514 |
| 15 | TOPIC: ("breast cancer") | 494,110 |
| 16 | TOPIC: ("prostatic neoplasms") | 6,102 |
| 17 | TOPIC: ("prostate cancer") | 196,850 |
| 18 | TOPIC: ("prostate neoplasms") | 823 |
| 19 | TOPIC: ("prostate neoplasm") | 358 |
| 20 | TOPIC: ("Zoledronic acid") | 8,632 |
| 21 | TOPIC: ("Zoledronate") | 1,649 |
| 22 | TOPIC: ("bone metastasis") | [8,331](https://apps-webofknowledge-com.proxy3.library.mcgill.ca/summary.do?product=WOS&doc=1&qid=22&SID=5Dr8CsPeKmUnMsPCPCt&search_mode=GeneralSearch&update_back2search_link_param=yes) |
| 23 | TOPIC: ("bone metastases") | [14,254](https://apps-webofknowledge-com.proxy3.library.mcgill.ca/summary.do?product=WOS&doc=1&qid=23&SID=5Dr8CsPeKmUnMsPCPCt&search_mode=GeneralSearch&update_back2search_link_param=yes) |
| 24 | TOPIC: ("bone mets") | [10](https://apps-webofknowledge-com.proxy3.library.mcgill.ca/summary.do?product=WOS&doc=1&qid=24&SID=5Dr8CsPeKmUnMsPCPCt&search_mode=GeneralSearch&update_back2search_link_param=yes) |
| 25 | #2 OR #1 | 61,204 |
| 26 | #6 OR #5 OR #4 OR #3 | 261,550 |
| 27 | #12 OR #11 OR #10 OR #9 OR #8 OR #7 | 14,742 |
| 28 | #15 OR #14 OR #13 | 496,326 |
| 29 | #19 OR #18 OR #17 OR #16 | 198,138 |
| 30 | #21 OR #20 | 9,853 |
| 31 | #24 OR #23 OR #22 | 20,267 |
| 32 | #29 OR #28 OR #27 OR #26 OR #25 | 953,690 |
| 33 | #32 AND #31 AND #30 | 1457 |

**Supplementary Table 4: Heterogeneity of the data when separating by primary cancer and time of administration for the different outcomes.** In the table, the odds ratios and the 95% credible intervals are shown for the skeletal-related events. For the time to new skeletal-related event, the progression-free survival and the overall survival, the hazard ratios with the corresponding 95% credible intervals are shown. For each measurement, the ***I squared*** **(I^2^)** is provided to describe the percentage of heterogeneity across studies. OR= Odds Ratio, HR= Hazard Ratio, CrI= Credible Intervals.

|  | **Skeletal-related event**  **OR (95% CrI), I^2^** | **Time to first skeletal-related event**  **HR (95% CrI), I^2^** | **Progression-free survival**  **HR (95% CrI), I^2^** | **Overall survival**  **HR (95% CrI), I^2^** |
| --- | --- | --- | --- | --- |
| **Primary cancer** | 1.76 (0.69,4.46), 2% | 1.86 (0.95, 1.93), 0% | 1.05 (0.89e-4, 4.72e12), 0% | 0.94 (0.44, 1.99), 8% |
| **Time of administration** | 0.81 (0.33, 1.96), 4% | 1.43 (0.88, 2.15), 3% | 1.07 (0.50, 1.70), 1% | 0.92 (0.49, 1.71), 9% |

|  | **Androgen blockade** | **Denosumab** | **Docetaxel** | **Everolimus** | **Other BP** | **Placebo** | **SR-89** | **ZA 4mg** | **ZA 8mg** | **ZA in combination** |
| --- | --- | --- | --- | --- | --- | --- | --- | --- | --- | --- |
| **Androgen blockade** |  |  |  |  |  |  |  |  |  |  |
| **Denosumab** | 0.67  (0.13, 3.57) |  |  |  |  |  |  |  |  |  |
| **Docetaxel** | 1.83  (0.44, 8.25) | 2.73  (0.55, 14.09) |  |  |  |  |  |  |  |  |
| **Everolimus** | 0.2  (0.01, 3.17) | 0.3  (0.01, 5.62) | 0.11  (0, 1.58) |  |  |  |  |  |  |  |
| **Other BP** | 0.34  (0.06, 1.96) | 0.51  (0.26, 1.03) | 0.19  (0.03, 0.99) | 1.69  (0.09, 66.13) |  |  |  |  |  |  |
| **Placebo** | 0.22  (0.04, 1.09) | 0.33  (0.18, 0.59) | 0.12  (0.02, 0.56) | 1.1  (0.06, 40.85) | 0.65  (0.35, 1.19) |  |  |  |  |  |
| **SR-89** | 0.28  (0.05, 1.61) | 0.41  (0.11, 1.46) | 0.15  (0.03, 0.86) | 1.37  (0.07, 57.39) | 0.81  (0.22, 2.84) | 1.25  (0.37, 4.25) |  |  |  |  |
| **ZA 4mg** | 0.37  (0.08, 1.89) | 0.55  (0.34, 0.89) | 0.2  (0.04, 0.95) | 1.8  (0.11, 73.13) | 1.08  (0.65, 1.79) | 1.65  (1.17, 2.52) | 1.34  (0.41, 4.62) |  |  |  |
| **ZA 8mg** | 0.36  (0.07, 1.92) | 0.52  (0.29, 0.96) | 0.19  (0.04, 0.96) | 1.74  (0.1, 70.03) | 1.03  (0.57, 1.87) | 1.59  (1.04, 2.49) | 1.28  (0.38, 4.59) | 0.96  (0.64, 1.4) |  |  |
| **ZA in combination** | 2.76  (1, 8.05) | 4.07  (1.18, 15.9) | 1.5  (0.55, 4.03) | 13.49  (1.13, 450.39) | 8.05  (2.24, 30.83) | 12.36  (3.74, 44.24) | 9.91  (2.37, 41.53) | 7.43  (2.21, 26.13) | 7.7  (2.23, 27.92) |  |

**Supplementary Table 5. Odds ratios of the different treatments when comparing their efficacy to decrease the number of SREs.**Inside the table, the odds ratios with the 95% credible intervals can be found. BP= Bisphosphonate, SR-89= Strontium-89, ZA= Zoledronic Acid.

**Supplementary Table 6. Hazard ratios of the different treatments when comparing their efficacy to increase the time to the first SRE.** Inside the table, the hazard ratios with the 95% credible intervals can be found. BP= Bisphosphonate, SR-89= Strontium-89, ZA= Zoledronic Acid.

|  | **Androgen Blockade** | **Denosumab** | **Docetaxel** | **Everolimus** | **Other BP** | **Placebo** | **SR-89** | **ZA 4mg** | **ZA 8mg** | **ZA Combined** |
| --- | --- | --- | --- | --- | --- | --- | --- | --- | --- | --- |
| **Androgen Blockade** |  |  |  |  |  |  |  |  |  |  |
| **Denosumab** | 4.36  (2.05, 9.19) |  |  |  |  |  |  |  |  |  |
| **Docetaxel** | 1.94  (0.9, 4.08) | 0.44  (0.19, 1.03) |  |  |  |  |  |  |  |  |
| **Everolimus** | 0.72  (0.25, 2.05) | 0.16  (0.05, 0.51) | 0.37  (0.12, 1.16) |  |  |  |  |  |  |  |
| **Other BP** | 3.16  (1.49, 6.61) | 0.73  (0.47, 1.11) | 1.63  (0.71, 3.83) | 4.39  (1.44, 13.5) |  |  |  |  |  |  |
| **Placebo** | 2.24  (1.15, 4.4) | 0.51  (0.35, 0.78) | 1.16  (0.54, 2.57) | 3.12  (1.07, 9.26) | 0.71  (0.48, 1.06) |  |  |  |  |  |
| **SR-89** | 3.3  (1.62, 6.65) | 0.76  (0.45, 1.27) | 1.7  (0.76, 3.86) | 4.59  (1.54, 13.7) | 1.04  (0.62, 1.75) | 1.47  (1, 2.14) |  |  |  |  |
| **ZA 4mg** | 3.88  (1.94, 7.64) | 0.89  (0.66, 1.2) | 2  (0.91, 4.44) | 5.39  (1.83, 15.9) | 1.23  (0.9, 1.66) | 1.73  (1.31, 2.25) | 1.17  (0.77, 1.79) |  |  |  |
| **ZA 8mg** | 3.11  (1.44, 6.65) | 0.71  (0.44, 1.17) | 1.6  (0.69, 3.84) | 4.32  (1.4, 13.58) | 0.98  (0.64, 1.51) | 1.38  (0.92, 2.06) | 0.94  (0.56, 1.61) | 0.8  (0.54, 1.19) |  |  |
| **ZA in combination** | 2.4  (1.51, 3.74) | 0.55  (0.3, 1) | 1.24  (0.68, 2.27) | 3.33  (1.28, 8.66) | 0.76  (0.42, 1.37) | 1.07  (0.64, 1.74) | 0.73  (0.42, 1.25) | 0.62  (0.37, 1.03) | 0.77  (0.41, 1.42) |  |

|  | **Androgen blockade** | **Denosumab** | **Docetaxel** | **Everolimus** | **Other BP** | **Placebo** | **SR-89** | **ZA 4mg** | **ZA 8mg** | **ZA in combination** |
| --- | --- | --- | --- | --- | --- | --- | --- | --- | --- | --- |
| **Androgen blockade** |  |  |  |  |  |  |  |  |  |  |
| **Denosumab** | 2.05  (0.91, 4.52) |  |  |  |  |  |  |  |  |  |
| **Docetaxel** | 1.52  (0.75, 3.08) | 0.74  (0.49, 1.14) |  |  |  |  |  |  |  |  |
| **Everolimus** | 0.82  (0.3, 2.26) | 0.4  (0.16, 1.04) | 0.54  (0.23, 1.28) |  |  |  |  |  |  |  |
| **Other BP** | 1.88  (0.82, 4.12) | 0.92  (0.63, 1.27) | 1.24  (0.78, 1.84) | 2.26  (0.87, 5.66) |  |  |  |  |  |  |
| **Placebo** | 2.12  (0.85, 5.05) | 1.04  (0.61, 1.68) | 1.4  (0.78, 2.37) | 2.57  (0.91, 6.82) | 0.65  (0.35, 1.19) |  |  |  |  |  |
| **SR-89** | 1.96  (0.9, 4.19) | 0.95  (0.75, 1.22) | 1.28  (0.92, 1.8) | 2.36  (0.94, 5.86) | 0.81  (0.22, 2.84) | 1.13  (0.77, 1.65) |  |  |  |  |
| **ZA 4mg** | 1.95  (0.85, 4.48) | 0.95  (0.63, 1.45) | 1.28  (0.8, 2.05) | 2.36  (0.9, 6.15) | 1.08  (0.65, 1.79) | 1.03  (0.84, 1.37) | 0.92  (0.6, 1.48) |  |  |  |
| **ZA 8mg** | 1.81  (0.96, 3.4) | 0.88  (0.52, 1.54) | 1.19  (0.84, 1.69) | 2.19  (1, 4.84) | 1.03  (0.57, 1.87) | 1.03  (0.71, 1.62) | 0.92   (0.54, 1.67) | 1  (0.71, 1.41) |  |  |
| **ZA in combination** | 2.05  (0.91, 4.52) | 0.74  (0.49, 1.14) | 0.54  (0.23, 1.28) | 2.26  (0.87, 5.66) | 8.05  (2.24, 30.83) | 0.96  (0.58, 1.72) | 0.85   (0.45, 1.69) | 0.93  (0.58, 1.52) | 0.93  (0.52, 1.68) |  |

**Supplementary Table 7. Hazard ratios of the different treatments when comparing their efficacy to increase the progression- free survival.** Inside the table, the hazard ratios with the 95% credible intervals can be found. BP= Bisphosphonate, SR-89= Strontium-89, ZA= Zoledronic Acid.

**Supplementary Table 8. Hazard ratios of the different treatments when comparing their efficacy to decrease the overall survival.** Inside the table, the hazard ratios with the 95% credible intervals can be found. BP= Bisphosphonate, SR-89= Strontium-89, ZA= Zoledronic Acid.

|  | **Androgen blockade** | **Denosumab** | **Docetaxel** | **Other BP** | **Placebo** | **SR-89** | **ZA 4mg** | **ZA in combination** |
| --- | --- | --- | --- | --- | --- | --- | --- | --- |
| **Androgen Blockade** |  |  |  |  |  |  |  |  |
| **Denosumab** | 1.07  (0.24, 4.75) |  |  |  |  |  |  |  |
| **Docetaxel** | 1.57  (0.39, 5.51) | 1.46  (0.47, 3.95) |  |  |  |  |  |  |
| **Other BP** | 0.99  (0.22, 4.38) | 0.92  (0.34, 2.48) | 0.63  (0.23, 1.95) |  |  |  |  |  |
| **Placebo** | 0.81  (0.21, 3) | 0.75  (0.31, 1.76) | 0.51  (0.22, 1.28) | 0.82  (0.33, 1.9) |  |  |  |  |
| **SR-89** | 0.75  (0.18, 2.94) | 0.69  (0.25, 1.88) | 0.48  (0.19, 1.33) | 0.75  (0.27, 2.04) | 0.93  (0.48, 1.81) |  |  |  |
| **ZA 4mg** | 1.12  (0.3, 4.16) | 1.04  (0.52, 2.1) | 0.71  (0.34, 1.68) | 1.13  (0.56, 2.28) | 1.39  (0.85, 2.36) | 1.5  (0.73, 3.14) |  |  |
| **ZA_in combination** | 1.28  (0.43, 3.8) | 1.19  (0.43, 3.3) | 0.82  (0.41, 1.84) | 1.3  (0.47, 3.6) | 1.59  (0.75, 3.53) | 1.72  (0.74, 4.1) | 1.15  (0.55, 2.41) |  |

|  | **Docetaxel** | **Other BP** | **Placebo** | **ZA 4mg** | **ZA 8mg** |
| --- | --- | --- | --- | --- | --- |
| **Docetaxel** |  |  |  |  |  |
| **Other BP** | -0.02  (-6.32, 6.76) |  |  |  |  |
| **Placebo** | 0.05  (-8.14, 8.20) | 0.09  (-6.65, 6.27) |  |  |  |
| **ZA 4mg** | -1.63  (-7.43, 4.23) | -1.63  (-4.86, 1.04) | -1.69  (-7.43, 4.05) |  |  |
| **ZA 8mg** | -0.74  (-8.96, 7.50) | -0.69  (-7.51, 5.58) | -0.79  (-6.63, 5.04) | 0.91  (-4.96, 6.73) |  |

**Supplementary Table 9: Level of pain at 3, 6 and 12 months after initiating the treatments.** Table showing the SMDs of the level of pain at A) 3 months, B) 6 months and C) 12 months after initiating the treatments. Inside the table, the SMDs with the 95% credible intervals can be found. BP= Bisphosphonate, ZA= Zoledronic Acid.

 B)

 A)

|  | **Other BP** | **Placebo** | **ZA 4mg** | **ZA 8mg** |
| --- | --- | --- | --- | --- |
| **Other BP** |  |  |  |  |
| **Placebo** | 0.38  (-0.75, 1.26) |  |  |  |
| **ZA 4mg** | -0.47  (-1.15, 0.07) | -0.85  (-1.59, -0.002) |  |  |
| **ZA 8mg** | 0.14  (-1.06, 1.26) | -0.25  (-1.20, 0.88)   C) | 0.60  (-0.35, 1.62) |  |

|  | **Docetaxel** | **Other BP** | **Placebo** | **ZA 4mg** | **ZA 8mg** |
| --- | --- | --- | --- | --- | --- |
| **Docetaxel** |  |  |  |  |  |
| **Other BP** | 0.11  (-3.38, 3.74) |  |  |  |  |
| **Placebo** | 1.90  (-1.88, 5.55) | 1.79  (-1.05, 4.34) |  |  |  |
| **ZA 4mg** | -0.73  (-3.82, 2.32) | -0.84  (-2.70, 0.85) | -2.64  (-4.65, -0.52) |  |  |
| **ZA 8mg** | 0.63  (-3.50, 4.72) | 0.52  (-2.83, 3.66) | -1.27  (-3.97, 1.58) | 1.35  (-1.41, 4.11) |  |
